# Causal associations between COVID-19 and childhood mental disorders

**DOI:** 10.1186/s12888-023-05433-0

**Published:** 2023-12-08

**Authors:** Fei Chen, Hongbao Cao, Ancha Baranova, Qian Zhao, Fuquan Zhang

**Affiliations:** 1 Department of Clinical Laboratory, The Affiliated Brain Hospital of Nanjing Medical University, Nanjing, 210029 China; 2 School of Systems Biology, George Mason University, Manassas, 20110 USA; 3 Research Centre for Medical Genetics, Moscow, 115478 Russia; 4 Department of Psychiatry, The Affiliated Brain Hospital of Nanjing Medical University, Nanjing, 210029 China; 5 Institute of Neuropsychiatry, The Affiliated Brain Hospital of Nanjing Medical University, Nanjing, 210029 China

**Keywords:** COVID-19, Attention-deficit/hyperactivity disorder, Mendelian randomization, Tourette’s syndrome, Autism spectrum disorder

## Abstract

**Background:**

The severe acute respiratory syndrome coronavirus 2 (SARS-CoV-2) can invade both the peripheral and central nervous systems and impact the function of the brain. Therefore, it is necessary to evaluate the mutual influences between COVID-19 outcomes and childhood mental disorders.

**Methods:**

We examined genetic correlations and potential causalities between three childhood mental disorders and three COVID-19 phenotypes by genetically proxied analyses. The three mental disorders included attention-deficit/hyperactivity disorder (ADHD, N = 292,548), Tourette’s syndrome (TS, N = 14,307), and autism spectrum disorder (ASD, N = 46,350). The three COVID-19 traits included SARS-CoV-2 infection (N = 2,597,856), hospitalized COVID-19 (N = 2,095,324), and critical COVID-19 (N = 1,086,211). Literature-based analysis was used to build gene-based pathways connecting ADHD and COVID-19.

**Results:**

ADHD was positively correlated with the three COVID-19 outcomes (R_g_: 0.22 ~ 0.30). Our Mendelian randomization (MR) analyses found that ADHD confers a causal effect on hospitalized COVID-19 (odds ratio (OR): 1.36, 95% confidence interval (CI): 1.10–1.69). TS confers a causal effect on critical COVID-19 (OR: 1.14, 95% CI: 1.04–1.25). Genetic liability to the COVID-19 outcomes may not increase the risk for the childhood mental disorders. Pathway analysis identified several immunity-related genes that may link ADHD to COVID-19, including *CRP*, *OXT*, *IL6*, *PON1*, *AR*, *TNFSF12*, and *IL10*.

**Conclusions:**

Our study suggests that both ADHD and TS may augment the severity of COVID-19 through immunity-related pathways. However, our results did not support a causal role of COVID-19 in the risk for the childhood mental disorders.

**Supplementary Information:**

The online version contains supplementary material available at 10.1186/s12888-023-05433-0.

## Introduction

COVID-19 has created a worldwide pandemic. It has been documented that the SARS-CoV-2 virus is neurotropic and neuroinvasive. In addition to the core symptoms from the respiratory system, neuropsychiatric manifestations are also common in COVID-19 patients [1–4]. Therefore, COVID-19 can adversely impact the brain function of patients, especially those with neuropsychiatric disorders [5–10].

With the ongoing spread of the pandemic, a significant proportion of infected individuals developed a variety of post-COVID symptoms, collectively known as “long COVID”. Among the various post-COVID complications, it has been reported that psychiatric patients are rising after the pandemic [11, 12], suggesting that coronavirus contributes to psychiatric symptoms and mental disorders through its ability to induce damage to neuron-glia homeostasis [13]. On the other hand, individuals with brain diseases may be more vulnerable to the impact of coronavirus, leading to severe outcomes after the infection.

A vital concern arises for children affected by the infection, considering the ongoing neurodevelopment and resultant vulnerability to the disturbances of the central nervous system (CNS) [14]. Earlier evidence showed that COVID-19 is less common in children and presents milder symptoms after the infection [15]. Despite this, COVID-19 severely affected children’s and adolescents’ mental health [16].

A recent antibody survey found that most (2/3) US children and adolescents aged 1–17 were exposed to the coronavirus [17]. The infection rate in children aged 1–4 exceeds those observed in adults during the Omicron wave [18]. Mental disorders, including those that occurred in childhood, are caused by a variety of neuroendocrine alterations, which may adversely influence the outcome of COVID-19. Autism spectrum disorder (ASD), Tourette’s syndrome (TS), and attention-deficit/hyperactivity disorder (ADHD) are three severe neurodevelopmental disorders that occur in children and possess a high heritability [19, 20]. Collectively, they may account for 15 ~ 30% of the disability-adjusted life-years, causing a substantial disability in this age group [21, 22]. They also have substantial phenotypic overlaps and shared genetic underpinnings with one another [23–28].

So far, evidence supporting associations between mental disorders and COVID-19 chiefly came from observational studies [29]. The causality between mental disorders and COVID-19 has yet to be explored. The Mendelian randomization (MR) framework infers potential causative associations between risk factors (exposures) and diseases (outcomes) by using genetic variants associated with exposure as instrumental variables [30]. The MR analysis is a widely used method to test causality between two traits [31, 32].

It’s not known whether the pathophysiological changes in children’s brains may exacerbate the process of COVID-19, or if childhood mental disorders could be triggered by outcomes of COVID-19. In this study, our objective was to assess the potential genetic connections between COVID-19 and three childhood mental disorders: ADHD, ASD, and TS. We hypothesize that specific genetic ties connect these mental health conditions and phenotypes of COVID-19. Gaining insights into these connections may contribute to improving both the management of COVID-19 and the care of individuals affected by these disorders.

## Methods

### GWAS summary datasets

Publicly available GWAS summary results on COVID-19 and three childhood mental disorders were used in this study. The three mental disorders included ADHD (38,691 cases and 275,986 controls) [33], ASD (18,381 cases and 27,969 controls) [34], and TS (4,819 cases and 9,488 controls) [35]. The summary GWAS datasets of COVID-19 were downloaded from the COVID-19 Host Genetics Initiative (HGI) (release on April 8, 2022), including critical COVID-19 (13,769 critically ill patients and 1,072,442 controls), hospitalized COVID-19 (32,519 hospitalized patients and 2,062,805 controls), and SARS-CoV-2 infection (122,616 virus-positive cases and 2,475,240 controls) [36]. All participants in the datasets were of European origin. Both critical COVID-19 and hospitalized COVID-19 were called “severe COVID-19” in this study.

### Genetic correlation analysis

The genetic correlations between the three mental disorders and the COVID-19 outcomes were assessed via linkage disequilibrium (LD) score regression [37, 38]. *P* values were adjusted by the false discovery rate (FDR < 0.05).

### MR analysis

The MR analysis has three assumptions on an instrumental variable (IV): (1) it needs to be associated with the exposure; (2) it cannot be associated with confounding factors influencing the exposure and the outcome; (3) it can only indirectly influence the outcome by its effect on the exposure [39]. Causal effects were inferred by three models in the TwoSampleMR package (version 0.5.6) [40], including the inverse variance weighting (IVW) model, the weighted median (WM) model, and the MR-Egger model, as complementary measures ensuring sensitivity [40, 41]. The IVW model operates under the assumption of zero intercepts and offers reliable estimates of causality via a fixed-effects meta-analysis approach. On the other hand, the MR-Egger model assumes that pleiotropic effects are independent and employs weighted linear regression to analyze outcome coefficients in relation to exposure coefficients. The pleiotropy is assessed by the intercepts of the MR-Egger regression [42]. If the MR-Egger intercepts significantly deviate from zero, it suggests that not all instrumental variables (IVs) are effective. The heterogeneity was evaluated by Cochran’s Q test and I^2^ statistics (*P* < 0.05 and I^2^ > 0.25). *P* values of the causal associations between COVID-19 and the mental disorders were adjusted by FDR (< 0.05). For each exposure phenotype, genome-wide significant (*P* < 5 × 10 ^–8^) SNPs (single nucleotide polymorphisms) associated with the exposure were pruned by a clumping r^2^ value of 0.01 within a 10 Mb window and used as IVs. When the IVs were less than 10, a relatively relaxed threshold of 1 × 10^− 5^ was used to pick IVs [43]. For the ASD and TS GWAS datasets, the threshold of 1 × 10^− 5^ was employed for IV selection.

### Literature-based analysis

To explore biological connections between ADHD and COVID-19, we utilized the Pathway Studio (www.pathwaystudio.com) environment to conduct literature-based data mining to create molecular pathways linking ADHD with COVID-19 [8, 44]. The Pathway Studio platform curated > 40 million scientific references, containing > 14 million unique associations. Initially, we identified the downstream targets and upstream regulators associated with both ADHD and COVID-19. Subsequently, we conducted a manual review of the references and each of the related sentences to ensure the quality of each extracted relationship. Relationships lacking polarity or those indirectly related to either COVID-19 or ADHD were eliminated. The remaining relationships were then utilized to construct a molecular pathway map that illustrates the connections between ADHD and COVID-19.

## Results

### Genetic correlation analysis

Our genetic correlation analyses showed that ADHD has significant positive genetic correlations with SARS-CoV-2 infection (r_g_ = 0.22 ± 0.05, *P* = 1.86E-06), hospitalized COVID-19 (r_g_ = 0.23 ± 0.04, *P* = 1.20E-07), and critical COVID-19 (r_g_ = 0.30 ± 0.05, *P* = 3.09E-09). ASD and TS did not display genetic correlations with the COVID-19 outcomes (Table 1).

**Table 1:** Genetic correlations between COVID-19 and three mental disorders

Trait 1	Trait 2	r_g_	se	Z	P	FDR
ADHD	Critical COVID-19	0.22	0.05	4.77	1.86E-06	5.57E-06
ADHD	Hospitalized COVID-19	0.23	0.04	5.29	1.20E-07	5.39E-07
ADHD	SARS-COV-2 infection	0.30	0.05	5.93	3.09E-09	2.78E-08
ASD	Critical COVID-19	-0.09	0.05	-1.70	0.090	0.202
ASD	Hospitalized COVID-19	-0.05	0.05	-0.988	0.323	0.485
ASD	SARS-COV-2 infection	-0.08	0.06	-1.23	0.220	0.396
TS	Critical COVID-19	0.01	0.06	0.154	0.877	0.987
TS	Hospitalized COVID-19	0.03	0.06	0.439	0.661	0.850
TS	SARS-COV-2 infection	0.00	0.08	0.008	0.993	0.993

ADHD: Attention-deficit/hyperactivity disorder; ASD: Autism spectrum disorder; TS: Tourette’s syndrome

### MR analysis

In the causal effect analysis of the mental disorders on the COVID-19 phenotypes, 26 IVs were yielded for ADHD (*P* < 5 × 10^–8^), 56–58 IVs for ASD (*P* < 1 × 10^–5^), and 36 IVs for TS (*P* < 1 × 10^–5^). The three COVID-19 datasets had different numbers of SNPs and the IVs were selected from the shared variants between an exposure and an outcome. Therefore, the numbers of IVs may vary across the three MR analyses, depending on a given exposure.

**Fig. 1:**
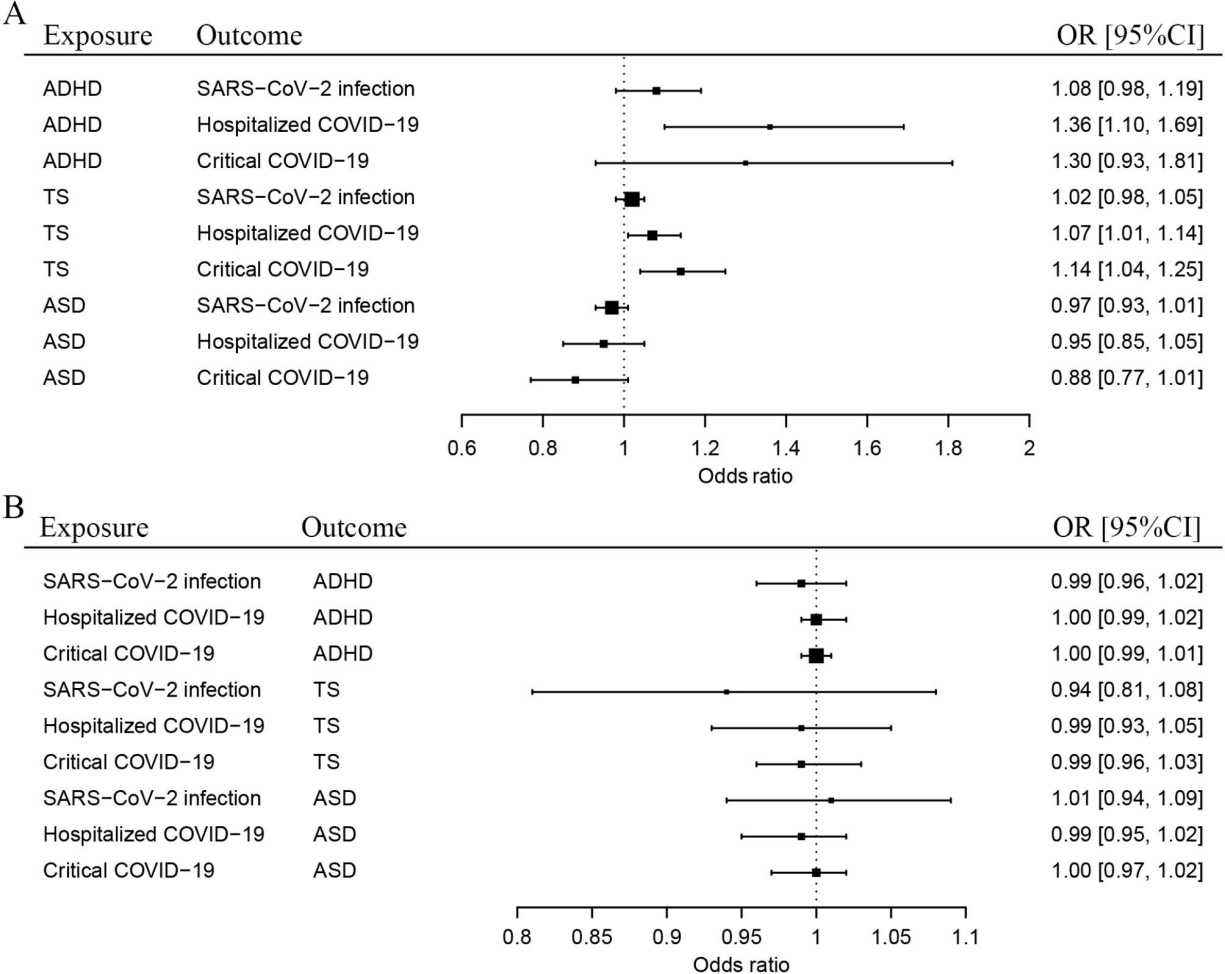
Bidirectional causal associations between COVID-19 and three mental disorders. **A**: Causal effects of three mental disorders on COVID-19 outcomes. **B**: Causal effects of the COVID-19 outcomes on three mental disorders

We found that ADHD confers a causal effect on hospitalized COVID-19 (OR: 1.36, 95% confidence interval (CI): 1.10–1.69). TS confers a causal effect on critical COVID-19 (OR: 1.14, 95% CI: 1.04–1.25) (Table 2; Fig. 1A). However, genetic liability to ASD did not have causal effects on the COVID-19 outcomes.

**Table 2:** Causal effects of three childhood mental disorders on COVID-19

Exposure	Outcome	b (se)	OR [95%CI]	N_IV	Q_P	I2	Egger_intercept	P_pleiotropy	P	FDR
ADHD	SARS-CoV-2 infection	0.077 (0.048)	1.08 [0.98–1.19]	26	0.342	0.083	0.003	0.46	0.108	0.162
ADHD	Hospitalized COVID-19	0.311 (0.108)	1.36 [1.10–1.69]	26	0.221	0.169	0.011	0.166	4.08E-03	0.020
ADHD	Critical COVID-19	0.261 (0.170)	1.30 [0.93–1.81]	26	0.128	0.245	0.004	0.754	0.126	0.162
TS	SARS-CoV-2 infection	0.016 (0.019)	1.02 [0.98–1.05]	36	8.97E-03	0.395	-0.001	0.788	0.388	0.388
TS	Hospitalized COVID-19	0.071 (0.031)	1.07 [1.01–1.14]	36	0.904	-0.42	-0.002	0.781	0.022	0.067
TS	Critical COVID-19	0.132 (0.047)	1.14 [1.04–1.25]	36	0.692	-0.153	0.006	0.493	4.47E-03	0.020
ASD	SARS-CoV-2 infection	-0.032 (0.020)	0.97 [0.93–1.01]	58	0.598	-0.06	-0.001	0.702	0.117	0.162
ASD	Hospitalized COVID-19	-0.055 (0.054)	0.95 [0.85–1.05]	57	4.35E-03	0.361	-0.005	0.474	0.312	0.351
ASD	Critical COVID-19	-0.123 (0.070)	0.88 [0.77–1.01]	56	0.196	0.137	-0.013	0.120	0.079	0.162

ADHD: Attention-deficit/hyperactivity disorder; ASD: Autism spectrum disorder; TS: Tourette’s syndrome; IVW: inverse variance weighted; WM: weighted median; OR: odds ratio; CI: confidence interval; N_IV: number of instrumental variables; Q_P: Cochran’s *P*-value of heterogeneity analysis

In the causal effect analysis of the COVID-19 conditions on the childhood mental disorders, we extracted 21–23 IVs for SARS-CoV-2 infection, 32–38 IVs for hospitalized COVID-19, and 32–37 IVs for critical COVID-19. We found that SARS-CoV-2 infection, critical COVID-19, and hospitalized COVID-19 have no causal effects on any of the mental disorders (Table 3; Fig. 1B).

**Table 3:** Causal effects of COVID-19 on three childhood mental disorders

Exposure	Outcome	b (se)	OR [95%CI]	N_IV	Q_P	I2	Egger_intercept	P_pleiotropy	P	FDR
SARS-CoV-2 infection	ADHD	-0.009 (0.017)	0.99 [0.96–1.02]	22	0.835	-0.423	-0.001	0.499	0.611	0.789
Hospitalized COVID-19	ADHD	0.002 (0.007)	1.00 [0.99–1.02]	32	0.106	0.246	0.001	0.358	0.776	0.789
Critical COVID-19	ADHD	0.001 (0.005)	1.00 [0.99–1.01]	32	0.085	0.267	0.001	0.696	0.777	0.789
SARS-CoV-2 infection	TS	-0.066 (0.072)	0.94 [0.81–1.08]	21	0.047	0.368	-0.008	0.288	0.364	0.789
Hospitalized COVID-19	TS	-0.013 (0.030)	0.99 [0.93–1.05]	38	4.27E-03	0.418	-0.008	0.200	0.677	0.789
Critical COVID-19	TS	-0.009 (0.019)	0.99 [0.96–1.03]	37	0.046	0.300	-0.008	0.172	0.644	0.789
SARS-CoV-2 infection	ASD	0.011 (0.037)	1.01 [0.94–1.09]	23	0.170	0.219	0.001	0.880	0.772	0.789
Hospitalized COVID-19	ASD	-0.015 (0.018)	0.99 [0.95–1.02]	36	4.06E-04	0.500	-0.002	0.580	0.416	0.789
Critical COVID-19	ASD	-0.003 (0.011)	1.00 [0.97–1.02]	36	4.29E-03	0.425	-0.001	0.875	0.789	0.789

ADHD: Attention-deficit/hyperactivity disorder; ASD: Autism spectrum disorder; TS: Tourette’s syndrome; IVW: inverse variance weighted; WM: weighted median; OR: odds ratio; CI: confidence interval; N_IV: number of instrumental variables; Q_P: Cochran’s *P*-value of heterogeneity analysis

The sensitivity analyses with different models indicated that the causal effects had the same directions across the three methods (Supplementary Tables 1–2). The MR-Egger result did not detect the pleiotropy in the MR analysis (MR-Egger intercept ≤ 0.011, *P* > 0.05). We found minimal evidence supporting the existence of heterogeneity, especially for the significant causal associations (Cochran’s *P* > 0.05 or I^2^ < 0.25).

### Literature-based analysis

**Fig. 2:**
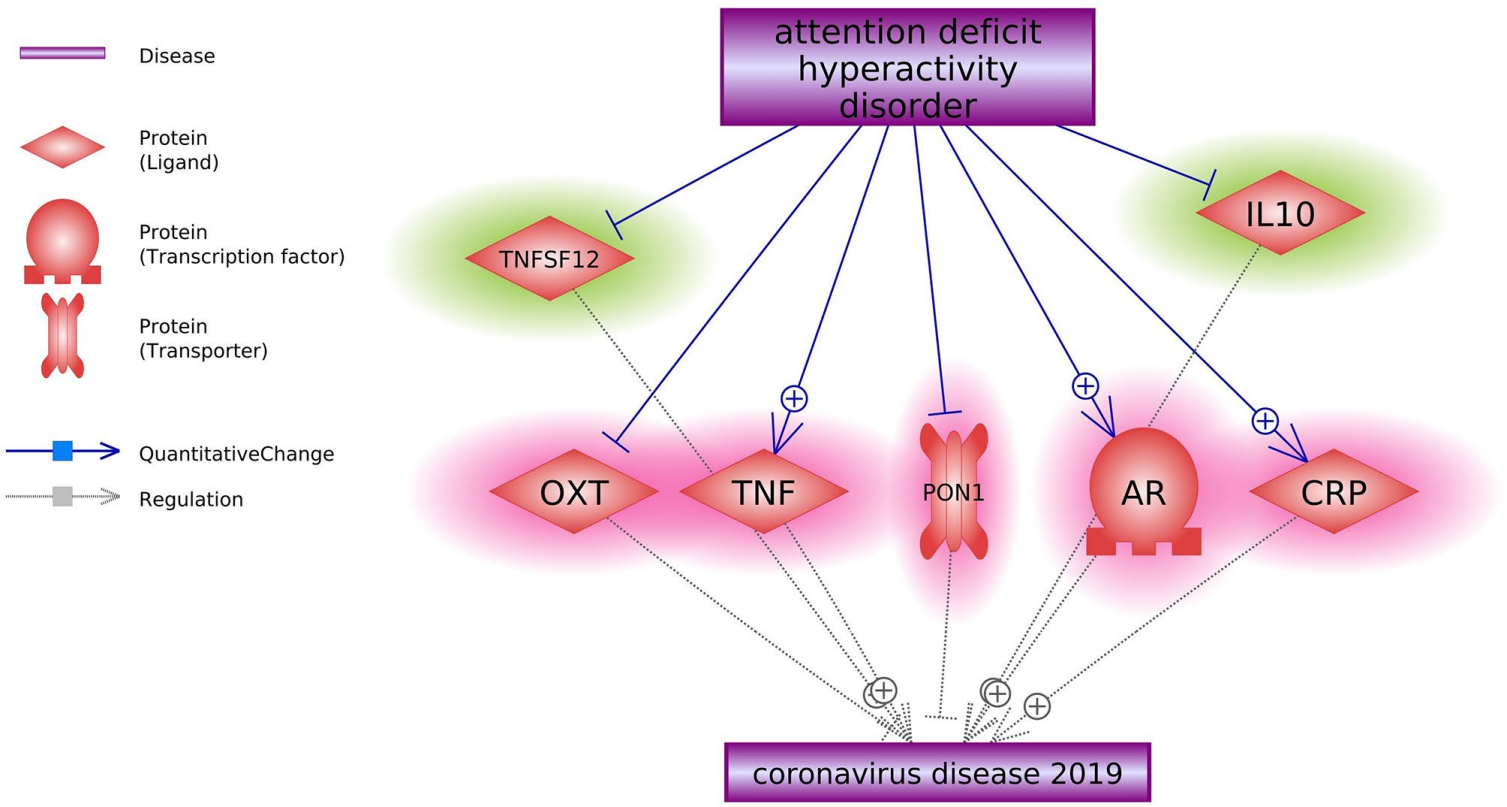
Literature-based molecular pathways connecting ADHD and COVID-19. Entities highlighted in green indicate a protective effect; Entities highlighted in red indicate a detrimental effect

Literature-based data mining and construction of the molecular pathways revealed a total of seven genes connecting ADHD with COVID-19, including *CRP*, *PON1*, *AR*, *OXT*, *IL6*, *TNFSF12*, and *IL10*. (Fig. 2). Among these connections, ADHD exerts a promotion effect on COVID-19 through the inhibition of *OXT* and *PON1* and the promotion of *CRP*, *AR*, and *TNF*.

## Discussion

Previous studies suggested that mental disorders and COVID-19 are mutual risk factors for one another, with the underlying mechanisms being largely unknown [45–47]. We carried out bidirectional MR analyses to detect causal connections between COVID-19 and three childhood mental disorders.

Our MR analysis provides robust evidence for the causal role of ADHD in the risk of COVID-19. Our results indicated that genetically determined ADHD was associated with a 36% enhanced risk for COVID-19 hospitalization. To date, only one MR study reported the causal influence of ADHD on COVID-19 hospitalization (OR: 1.297 [1.029–1.634], *P* = 0.028) [48]. Our results corroborated the previous finding with more robust associations using the largest ADHD dataset and the largest COVID-19 datasets. The positive genetic correlations between ADHD and the COVID-19 phenotypes provide additional evidence for their close relationship. It was suggested that ADHD seems to constitute a behavioral risk factor both for SARS-CoV-2 infection and for severe outcomes of COVID-19 [49].

For TS, our findings showed that TS was associated with a 14% increased risk for critical COVID-19. Our results support that ADHD and TS may exacerbate the pathophysiology of COVID-19. Although TS has the highest comorbidity rate with ADHD [25], studies on the TS-COVID-19 connection were sparse. Therefore, we focused on ADHD for further discussion.

In the case of ASD, a 40% longer SARS-CoV-2-related mean hospital stays were noted [50], and a recent study also showed that COVID-19 has had psychological effects and led to increased difficulties among children with ASD [51]. However, our study did not support causal associations between ASD and COVID-19 outcomes in the context of genetic underpinning. ADHD and ASD co-occur commonly and have phenotypic overlaps and shared genetic components [24, 28, 52–54]. The two disorders also have different genetic properties, including their opposite genetic correlations with intelligence [55].

As a vital adverse exposure, COVID-19 pandemics aggravated individual life trajectories both in patients infected with SARS-CoV-2 and in virus-naïve bystanders exposed to pandemic-related stress, likely contributing to the accumulation of mental diagnoses in the general population. The unfavorable influences of COVID-19 on ADHD and ASD have been well documented [56–58]. In this study, our study indicated that COVID-19 may not be associated with the risk for the childhood mental disorders. Therefore, the increased symptoms of ADHD and ASD associated with COVID-19 may presumably be due to socio-psychological or environmental aspects of the pandemic [56].

To explore possible mechanisms underlying their connection, we created gene-based pathways linking ADHD and COVID-19. The constructed pathways support this MR analysis result at the molecular level. SARS-CoV2 infection has been shown to promote tumor necrosis factor-alpha (TNF-α) expression and secretion [59]. In addition, C-reactive protein (CRP) was frequently found elevated in COVID-19 patients [60], including children with severe MIS-C [61]. Studies have shown that increased CRP levels are positively correlated with the severity of COVID-19 [62]. Interestingly, non-critical COVID-19 patients were found with a significantly increased level of serum adrenocorticotropic hormone (ACTH) [63]. Notably, the levels of ACTH differentiate non-critical COVID-19 patients from non-critical ones [63, 64], possibly pointing at pre-existing or developing adrenal insufficiency associated with a severe form of COVID-19 [65]. On the other hand, in ADHD patients, the HPA axis may be under-reactive, thus, predisposing them to the severity of the coronavirus disease. Moreover, recombinant ACTH has been reported to improve symptom severity in ADHD [66].

Patients with ADHD commonly present with significantly decreased concentrations of oxytocin (encoded by *OXT*) [67]. Coupaye et al’s work showed that the administration of oxytocin may impede the progression to severe COVID-19 by suppressing cytokine storm and blocking viral invasion [68]. Therefore, oxytocin has been suggested as a candidate for treating COVID-19 [69]. By the depressing expression of *OXT*, ADHD may increase the propensity of an individual to develop severe COVID-19 and to be hospitalized [70]. In addition, the role of CRP in ADHD was also suggested [71]. This implies that ADHD-related increases in systemic CRP concentrations [72] may play a role in pulmonary fibrosis in COVID-19 [73]. To sum up, the investigation of molecular pathways supported our MR-facilitated inference of the causal effects of genetic liability to ADHD on severe COVID-19. These conclusions are consistent with the clinical observation that ADHD was associated with poorer outcomes after the SARS-CoV-2 infection [49].

A limitation of this study is the omission of the records of medication, potentially introducing unaccounted variability into the dataset. For a comprehensive understanding, the results of the current MR study should be taken into account alongside available clinical evidence. An additional constraint is that MR analysis solely investigates the causal relationship at the genetic level. To comprehend the comprehensive connections between COVID-19 and mental health disorders, it is essential to consider psychosocial factors and environmental variables as potential mediators. Lastly, direct laboratory data is essential to corroborate the literature-based molecular pathway.

## Conclusions

In summary, our study showed that both ADHD and TS may aggravate the severity of COVID-19, while COVID-19 may not directly contribute to the risk of the childhood mental disorders.

### Electronic supplementary material

Below is the link to the electronic supplementary material.

Supplementary Material 1

Supplementary Material 2

## Data Availability

All de-identified data, including individual participant data, are publicly available. The COVID-19 datasets were available in the COVID-19 Host Genetics Initiative (https://www.covid19hg.org/results/r7/). The datasets for the three mental disorders were available in the Psychiatric Genomics Consortium (https://pgc.unc.edu/for-researchers/download-results/).
